# Brain volumetric analysis of elderly patients based on hearing levels quantified using FreeSurfer

**DOI:** 10.3389/fneur.2025.1602383

**Published:** 2025-07-23

**Authors:** Yun Ji Lee, Eun-Jae Lee, Chong Hyun Suh, Hye Jung Youn, Jihye Chae, Keunwoo Park, Jihoon Kweon, Joong Ho Ahn

**Affiliations:** ^1^Department of Otorhinolaryngology-Head and Neck Surgery, Asan Medical Center, University of Ulsan College of Medicine, Seoul, Republic of Korea; ^2^Department of Neurology, Asan Medical Center, University of Ulsan College of Medicine, Seoul, Republic of Korea; ^3^Department of Radiology and Research Institute of Radiology, Asan Medical Center, University of Ulsan College of Medicine, Seoul, Republic of Korea; ^4^Department of Convergence Medicine, Asan Medical Center, University of Ulsan College of Medicine, Seoul, Republic of Korea

**Keywords:** hearing loss, gray matter, limbic system, hippocampus, MRI, FreeSurfer

## Abstract

**Objective:**

This study investigated the effects of hearing loss on brain volume, particularly total gray matter, the cerebellum, and the limbic system, using MRI data analyzed with FreeSurfer software.

**Study Design:**

Data was obtained from 745 individuals aged 60 or older who underwent health screenings at Asan Medical Center between March and December 2019. Participants were categorized into three groups based on their pure-tone hearing thresholds: normal hearing (<20 dB, *n* = 328), mild hearing loss (20–40 dB, *n* = 336), and moderate-to-severe hearing loss (≥40 dB, *n* = 81).

**Results:**

Results showed significant differences among the groups in age, sex, and health conditions such as hypertension, diabetes, and cholesterol levels (*p* < 0.05). Increased hearing loss severity correlated with older age, male predominance, and higher prevalence of these conditions. Multiple regression analysis, controlling for confounders like age and comorbidities, revealed age-related brain atrophy across all groups. Notably, even mild hearing loss was associated with reduced total gray matter volume, while moderate-to-severe hearing loss led to significant hippocampal volume reduction.

**Conclusion:**

The findings suggest that hearing loss in individuals over the age of 60 may contribute to the atrophy of critical brain regions, particularly the hippocampus and total gray matter. The study emphasizes the importance of addressing even mild hearing loss, as active hearing rehabilitation could potentially prevent cortical volume loss and mitigate the risk of cognitive decline.

## Introduction

Contemporary research has revealed a significant association between hearing loss and cognitive function as well as the onset of dementia. Therefore, there is growing interest in understanding how the mechanisms responsible for acquired hearing loss affect the higher-level auditory system (1, 2). This has become technically feasible thanks to advanced image processing software, which enables the evaluation of multiresolution cortical thickness and volume estimation (3). FreeSurfer is an open-source software suite for processing and analyzing human brain MRI images (4). It does so by visualizing structural and functional neuroimaging data from cross-sectional or longitudinal studies and provides brain segmentation, labeling of regions, and permits the statistical analysis of group morphometry differences.

Several studies have examined the relationship between hearing loss and brain volume, including the primary auditory cortex, in the adult population (5, 6). A longitudinal analysis of auditory cortex morphology in age-related hearing loss (ARHL) revealed a decrease in gray matter (GM) volume in the primary auditory cortex (7). In another study, elderly individuals with elevated pure-tone thresholds experienced a more significant change in GM volume (8). On the other hand, when using MR morphometry and diffusion tensor imaging to elucidate the central component of ARHL, some findings have indicated that the severity of hearing loss did not have a significant impact (9).

Various studies have explored the impact of hearing loss on different areas of the brain. In one example, researchers conducted functional MRI scans of a group of individuals aged over 60 with normal hearing, and suggested a potential association between the cortical speech processing network and higher-level cognitive functions (10). However, the causality of hearing loss concerning cognitive functions is not yet fully understood. Here, we aim to elucidate the extent of volume changes in specific brain regions in response to varying severity of hearing impairment. In particular, we focused on areas responsible for memory and cognitive functions.

## Materials and methods

### Study participants

**Figure 1:**
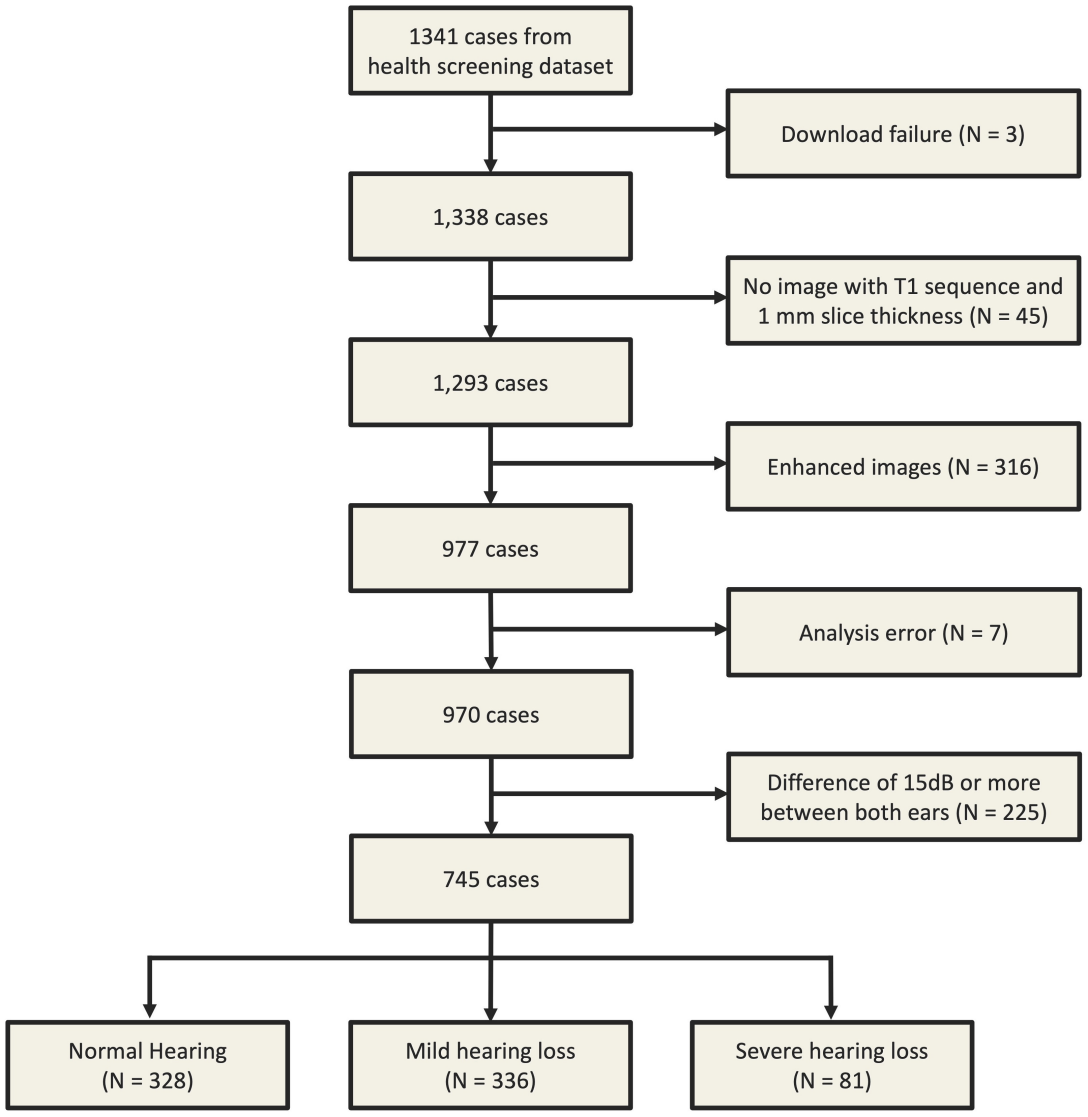
From 1,341 data requests, 1,338 datasets were downloaded, and after filtering and verifying T1 images, 970 files remained. Analyses focused on 745 patients, categorized into three subgroups based on hearing levels.

This study used data from a health screening dataset created by Asan Medical Center from March to December 2019. During this period, data for 1,341 subjects aged ≥60 years was obtained. Of the 1,341 participants, 970 cases were included for which brain MRI data could be obtained. Here we excluded cases with download failures (*n* = 3), analysis errors (*n* = 7), missing T1 images or images with a thickness other than 1 mm (*n* = 45), and cases with enhanced images (*n* = 316) since these were not suitable for FreeSurfer analysis. We also excluded cases in which there was an inter-ear difference in hearing of more than 15 dB (*n* = 225; Figure 1). Finally, demographic features of patients, including blood lipid levels (i.e., LDL, HDL, triglycerides, and total cholesterol), hypertension (HTN), diabetes (DM), glucose, and HbA1C levels, were examined. In addition, a survey on smoking and alcohol consumption habits was conducted.

### Brain MRI and cortical volumetry measurements

**Figure 2:**
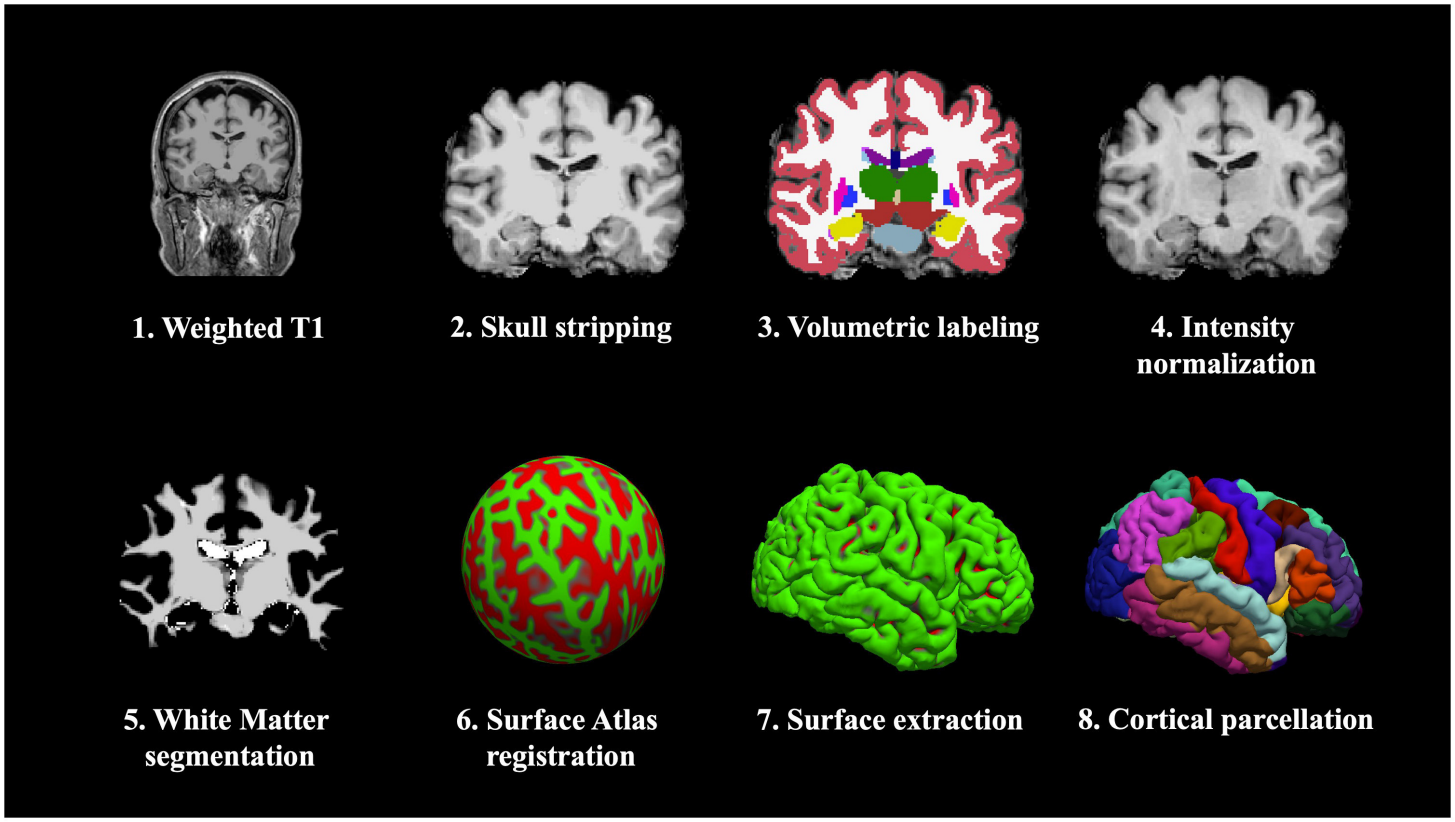
Brain MRI analysis process using FreeSurfer.

MR images were obtained using a 3.0-T MRI scanner (Achieva 3T TX MRI System, Philips HealthCare, Best, Netherlands). T1-weighted imaging was performed in the sagittal orientation with the following parameters: TR/TE = 500/10; FA = 90°; FOV = 240; matrix = 512 × 512; thickness = 1 mm. Cortical surface reconstructions and regional GM volume estimations were performed using FreeSurfer version 7.2.0 (Figure 2).

In this process, raw Digital Imaging and Communications in Medicine (DICOM) files were first converted to NIfTI (Neuroimaging Informatics Technology Initiative) format, followed by registration to the MNI305 template. Initial image preprocessing steps such as bias field correction, skull stripping, and tissue segmentation were applied. The hemispheres were then separated, and white and pial surfaces were reconstructed along intensity gradients to enable accurate cortical modeling. The procedural details of these analyses have been documented in prior publications (11–14). For cortical regions, FreeSurfer uses the Desikan–Killiany atlas to parcellate regions of interest based on cortical gyri (15, 16). In addition, subcortical structures were automatically segmented and labeled, following the methodology presented by Fischl et al. (14). The analyzed regions of interest included the total GM, limbic system, including the hippocampus, thalamus, amygdala, accumbens, as well as the white matter and cortex of the cerebellum for both hemispheres.

### Otologic examination and audiologic evaluation

Audiological evaluation was performed using a diagnostic audiometer (SA-203; Entomed, Malmö, Sweden) at 0.5, 1, 2, and 4 kHz in a soundproof booth. The average thresholds for each of the four frequencies for each ear were used. Moreover, cases with a history of sudden hearing loss, Meniere's disease, or other inner ear disorders were excluded. We included patients with bilateral symmetric hearing thresholds (within a threshold difference of 15 dB), which is consistent with the definition of ARHL. Patients with no significant abnormalities found in the tympanic membrane examination and no history of otitis media or other related conditions were included in this study. The analysis was performed by categorizing the hearing levels of both ears into three groups: < 20 dB, 20–40 dB, and above 40 dB.

The study protocol for data analysis was approved by the Institutional Review Board (IRB 2020-0646). The studies were conducted in accordance with the local legislation and institutional requirements. Written informed consent for participation was not required from the participants or the participants' legal guardians/next of kin in accordance with the national legislation and institutional requirements.

### Statistical analysis

We used Kruskal–Wallis tests and Fisher's exact tests to compare continuous- and categorically-measured demographic characteristics among the subgroups, respectively. Next, we adopted an approach employed in a previous study such that each specific regional volume was adjusted by dividing it by the estimated total intracranial volume then multiplying this result by the average estimated total intracranial volume for all subjects (to accommodate variation in head size among individuals) (17). The resulting estimates were represented in cubic millimeters (mm^3^).

To ensure the statistical robustness of our findings, a priori power analysis was conservatively performed assuming a small effect size (Cohen's *f*^2^ = 0.10), a stringent significance level (α = 0.01), and a high power threshold (1–β = 0.99). Under these criteria, the minimum required sample size for a multiple regression model with six predictors (Age, Sex, HTN, DM, Hyperlipidemia, Hearing group) was estimated to be 203. Since the total sample size in this study far exceeded this threshold, the analysis was sufficiently powered even under these conservative conditions.

Multivariable linear regression analyses were used to confirm the association between hearing impairment and volumetric changes in the brain. To control for potential confounding factors, covariates including age (continuous), sex (binary), and the presence of HTN, DM, and hyperlipidemia (all coded as binary variables: 0 = absence, 1 = presence) were included in the model. All variables were entered using stepwise method. To assess multicollinearity among the independent variables, the independence of residuals was evaluated using the Durbin-Watson statistics. In all models, tolerance values were >0.1, variance inflation factors were below 5, and condition indices were < 15, indicating no substantial multicollinearity. Residual normality was assessed using the Shapiro–Wilk test and Q–Q plots. The results indicated no significant deviation from normality (*p* > 0.05). For all analyses, a *p*-value < 0.05 was considered to be statistically significant. All statistical analyses were performed using SPSS version 29.0 (IBM SPSS Inc.).

## Results

### Demographic characteristics of the subjects were stratified by hearing level

**Figure 3:**
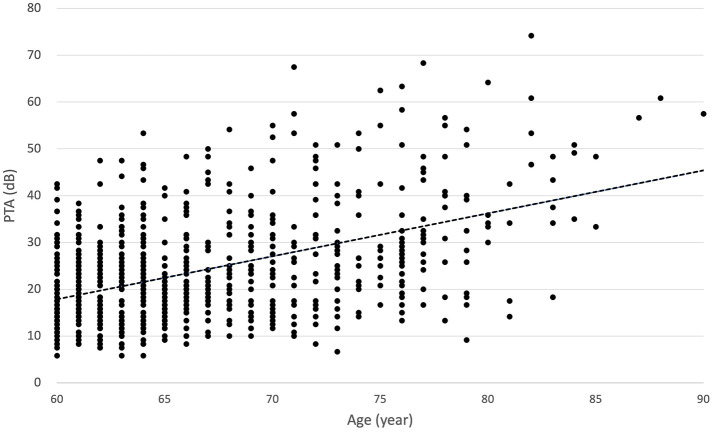
According to the linear regression analysis, there was a statistically significant increase in the average pure-tone audiometry results for both ears as patient age increased (*B* = 0.94, *r*^2^ = 0.22, *p* < 0.000).

The baseline characteristics of study participants according to hearing level are shown in Supplementary Table 1. A total of 745 patients were included. Our sample showed a median age of 65 years and 405 (54.36%) of all subjects were male. When divided into three groups based on hearing level, we observed that the severity of hearing loss was associated with older patients, as well as a higher proportion of males (*p* < 0.05). When examining the relationship between average PTA and patient age via linear regression, it was observed that as age increased, the hearing threshold significantly increased (*B* = 0.94, *r*^2^ = 0.22, *p* < 0.000; Figure 3).

Patients in moderate-to-severe hearing loss showed significantly higher prevalence in HTN, DM, and showed higher level of glucose and HbA1c than patients in normal hearing and mild hearing loss (Supplementary Table 1). Contrary to our expectations, the total cholesterol and LDL levels were significantly lower in the worse hearing group, despite their higher average age. There was a slight difference in smoking history, with a higher percentage of current smokers in the moderate-to-severe hearing loss group, but this difference was not statistically significant.

### Brain volumetric comparisons of auditory groups

**Figure 4:**
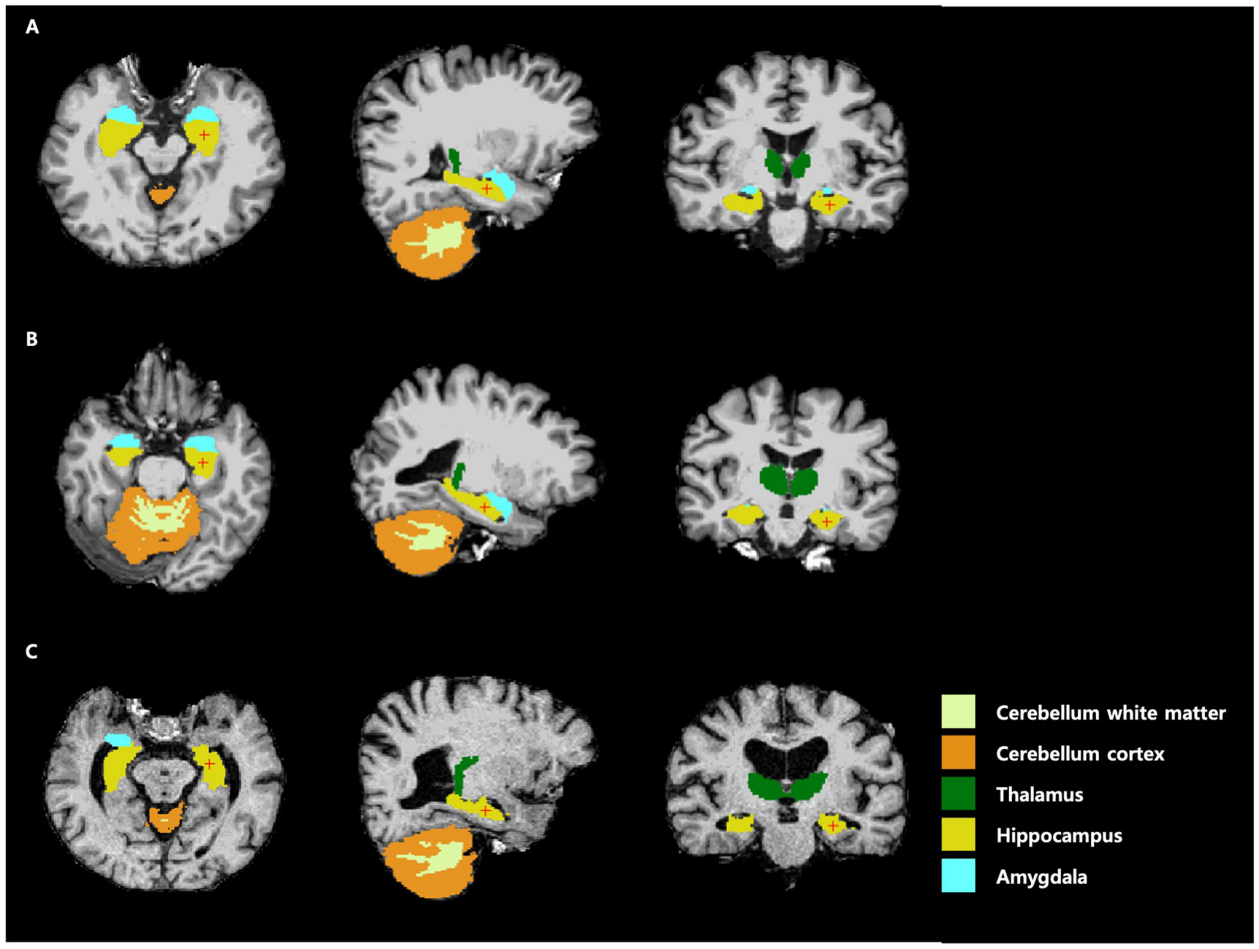
Anatomical regions of interest for analysis using FreeSurfer. **(A)** Normal hearing, (**B)** mild hearing loss, and **(C)** moderate-to-severe hearing loss.

Next, we compared cerebellar white matter, cerebellar cortex, limbic system components, and total GM volume among the three subgroups (Supplementary Table 2, Figure 4). The more severe the degree of hearing loss, the greater the tendency for most areas to show a significant decrease in volume (*p* < 0.01). There was a significant decrease in the volume of the bilateral cerebellar white matter, cerebellum cortex, thalamus, hippocampus, amygdala, accumbens, and total GM in the moderate-to-severe hearing loss group compared to normal hearing. However, there were no significant differences among groups with respect to the size of the caudate and pallidum.

### Multiple regression analyses of the relationship between hearing level and brain volume

We examine the relationship between hearing loss severity and brain volume through multiple regression analysis. After adjusting for factors such as age, sex, and medical history of HTN, DM, and hyperlipidemia, multiple regression analysis was conducted (Table 1). In the areas analyzed, cortical volume significantly decreased with age (*p* < 0.001). A significant volume reduction in both hippocampi was observed in the moderate-to-severe hearing loss group (*p* < 0.05), although there was no significant difference between the normal hearing and mild hearing loss group. Total GM volume reduction was observed in the mild hearing loss group, and this trend was even more pronounced in the moderate-to-severe hearing loss group. However, no meaningful volume changes were observed in the white matter of the cerebellum, the cerebellar cortex, and other areas of the limbic system (i.e., thalamus, amygdala and accumbens) among the groups.

**Table 1:** Multiple regression analyses of the relationship between hearing level and brain volume.

**Segmentation area (mm^3^)**	***r* ^2^**	**Mild hearing loss (*****n*** = **336)**			**Moderate-to-severe hearing loss (*****n*** = **81)**		
		**Standardized coefficient**	***t***	***p*-value**	**Standardized coefficient**	***t***	***p*-value**
**Total Gray matter**	**0.224**	**−0.077**	**−2.170**	**0.030**	**−0.106**	**−2.761**	**0.006**
**Left**							
Cerebellar white matter	0.194	0.016	0.455	0.649	0.007	0.168	0.867
Cerebellum Cortex	0.097	−0.059	−1.539	0.124	−0.059	−1.430	0.153
Thalamus	0.199	0.012	0.339	0.735	0.077	1.983	0.051
**Hippocampus**	0.234	−0.066	−1.873	0.061	**−0.080**	**−2.091**	**0.037**
Amygdala	0.110	−0.040	−1.060	0.290	−0.039	−0.944	0.345
Accumbens	0.156	−0.070	−1.885	0.060	−0.065	−1.622	0.105
**Right**							
Cerebellar white matter	0.186	0.08	0.486	0.627	−0.025	−0.632	0.528
Cerebellum Cortex	0.082	−0.071	−1.855	0.064	−0.047	−1.125	0.261
Thalamus	0.169	−0.003	−0.078	0.938	0.038	0.954	0.341
**Hippocampus**	0.236	−0.068	−1.937	0.053	**−0.087**	**−2.283**	**0.023**
Amygdala	0.084	−0.014	−0.370	0.712	−0.041	−0.980	0.328
Accumbens	0.129	−0.034	−0.919	0.359	−0.069	−1.686	0.092

The analysis was adjusted for age, sex, and medical history (hypertension, diabetes, and hyperlipidemia), and presents standardized coefficients and *p*-values for each brain region. Across all categories, brain volume decreased with increasing age, and males exhibited significantly larger volumes (*p* < 0.001).

Statistically significant results (*p* < 0.05) are shown in bold.

Multiple linear regression analyses were performed separately for 13 cortical regions, with hearing groups as the main predictor. To correct multiple comparisons across the 13 regions, the significance of the hearing group factor was assessed using Type III sum of squares within the general linear model framework. The resulting *p*-values were adjusted using the false discovery rate (FDR) method based on the Benjamini–Hochberg procedure. Although uncorrected *p*-values ranged from 0.011 to 0.902, none reached the predefined significance threshold after FDR correction (Supplementary Table 3).

## Discussion

This study used health examination data from a single tertiary center to reveal that significant volume changes in brain MRI were related to hearing loss in elderly Koreans aged over 60. For example, when comparing groups based on the degree of hearing loss, we observed that the moderate-to-severe hearing loss group was significantly older and had a higher proportion of males, reflecting the demographic features known in ARHL. After minimizing the influence of other factors that could cause changes in the brain cortex, it was found that the volume of the bilateral hippocampi and total GM were significantly smaller in the hearing loss group relative to the normal hearing group.

Consistent with the findings of this study were data from a study that identified a relationship between hippocampal atrophy and hearing loss in the middle-aged population in Japan (17). On the other hand, there have also been reports indicating that the impact of hearing loss on hippocampal volume is limited, whereas a significant increase was observed due to tinnitus (18). In most forms of dementia, the hippocampus is one of the earliest-affected structures (19, 20). In patients with dementia, the hippocampus typically undergoes significant changes associated with memory impairment. As dementia progresses, the hippocampus further shrinks, thereby significantly affecting memory and learning abilities. We suggest that volume reduction in this area due to hearing loss could be related to memory decline and therefore suggest that this mechanism may cause cognitive dysfunction.

The cerebellum is known to be involved in specific cognitive functions, which suggests its potential involvement in the cognitive and neuropsychiatric decline observed in dementia (21). Nevertheless, the precise function of the cerebellum in the development of Alzheimer's disease remains uncertain (22). The distinctive histopathological findings of Alzheimer's disease, including accumulation of extracellular amyloid β (Aβ) plaques and intracellular neurofibrillary tangles, are known to occur in the cerebellum only at advanced stages of the disease. When examining the volume changes in the cerebellar white matter and cerebellar cortex, we observed that individuals with moderate-to-severe hearing loss tended to have smaller volumes. However, after adjusting for age and conducting a multiple regression analysis, we revealed that no significant differences were found among the groups. When excluding the influence of age, which is a factor causing significant volume reduction, it was confirmed that there were no cerebellar volume changes due to hearing loss.

Both brain cortical atrophy occurring during the typical aging process and pathological alterations often seen in dementia have been well documented. In relation to aging, cortical atrophy due to GM loss occurs linearly (18, 23). In patients with AD, a gradual decrease in GM extends from the temporal and limbic cortices to the frontal and occipital regions of the brain (24). In our study, we found that total GM volume significantly decreased with age. After correcting this factor and examining for significant differences among the hearing loss groups, we observed a progressive reduction in volume, particularly in the mild and moderate-to-severe hearing loss groups.

Midlife hearing loss may contribute to dementia through multiple mechanisms—including reduced cognitive reserve, increased cognitive load during listening, and interactions between altered auditory processing in the medial temporal lobe and Alzheimer's pathology—highlighting hearing loss as a potentially modifiable risk factor (25). In line with previous research, the present study offers direct evidence supporting the role of hearing loss as a contributing factor to cognitive decline and the advancement of dementia.

This study was a cross-sectional study and did not analyze longitudinal brain volumetric changes; therefore, we could not establish a causal relationship with hearing loss. Moreover, we focused on the limbic system, cerebellum, and total GM; however, we note that there may be other brain areas affected by hearing loss that were not explored. In addition, there could be other confounding factors that influence the brain cortex. In addition, the absence of data regarding whether participants with hearing loss were undergoing auditory rehabilitation, such as the use of hearing aids, represents a limitation of this study. In future studies, it would be beneficial to include additional potential confounding factors such as auditory rehabilitation (e.g., hearing aid use), cognitive function, educational level, and social engagement, as these may influence the outcomes.

Although multiple linear regression analyses revealed nominally significant associations between hearing group and cortical volume in certain brain regions, none of these findings survived correction for multiple comparisons using the FDR method. This suggests that the observed effects may not be robust at the statistical level after controlling for potential false positives across the 13 cortical regions. Nevertheless, the consistent trends observed—hippocampus and total GM—support the biological plausibility of our findings. Given the exploratory nature of this study and the known sensitivity of neuroimaging results to conservative statistical thresholds, these preliminary findings may still provide meaningful insights into how hearing loss severity may relate to brain morphology. Further studies with larger sample sizes and longitudinal designs are warranted to confirm these associations and clarify their clinical implications.

Nevertheless, despite these limitations, in this study an analysis of a large number of patients using multiple regression analysis revealed a significant decrease in hippocampal volume in the moderate-to-severe hearing loss group that was not observed in the mild hearing loss group. Furthermore, we also observed that the volume of total GM progressively decreased more significantly with higher degree of hearing loss. Taken together, these findings may be crucial for understanding the relationship between hearing loss and cognitive ability.

## Data Availability

The original contributions presented in the study are included in the article/Supplementary material, further inquiries can be directed to the corresponding author.

## References

[1] Lin FR Yaffe K Xia J Xue QL Harris TB Purchase-Helzner E. Hearing loss and cognitive decline in older adults. JAMA Intern Med. (2013) 173:293–9. 10.1001/jamainternmed.2013.1868 23337978 PMC3869227

[2] Livingston G Sommerlad A Orgeta V Costafreda SG Huntley J Ames D. Dementia prevention, intervention, and care. Lancet. (2017) 390:2673–734. 10.1016/S0140-6736(17)31363-6 28735855

[3] Nian R Gao M Zhang S Yu J Gholipour A Kong S. Toward evaluation of multiresolution cortical thickness estimation with freesurfer, macruise, and brainsuite. Cereb Cortex. (2023) 33:5082–96. 10.1093/cercor/bhac401 36288912

[4] Fischl B. Freesurfer. Neuroimage. (2012) 62:774–81. 10.1016/j.neuroimage.2012.01.021 22248573 PMC3685476

[5] Rigters SC Bos D Metselaar M Roshchupkin GV Baatenburg de. Jong RJ, Ikram MA, et al. Hearing impairment is associated with smaller brain volume in aging. Front Aging Neurosci. (2017) 9:2. 10.3389/fnagi.2017.00002 28163683 PMC5247429

[6] Neuschwander P Hänggi J Zekveld AA Meyer M. Cortical thickness of left Heschl's gyrus correlates with hearing acuity in adults - a surface-based morphometry study. Hear Res. (2019) 384:107823. 10.1016/j.heares.2019.107823 31678891

[7] Eckert MA Vaden KI Jr Dubno JR. Age-related hearing loss associations with changes in brain morphology. Trends Hear. (2019) 23:2331216519857267. 10.1177/2331216519857267 31213143 PMC6585256

[8] Lin FR Ferrucci L An Y Goh JO Doshi J Metter EJ. Association of hearing impairment with brain volume changes in older adults. Neuroimage. (2014) 90:84–92. 10.1016/j.neuroimage.2013.12.059 24412398 PMC3951583

[9] Profant O Škoch A Balogová Z Tintěra J Hlinka J Syka J. Diffusion tensor imaging and MR morphometry of the central auditory pathway and auditory cortex in aging. Neuroscience. (2014) 260:87–97. 10.1016/j.neuroscience.2013.12.010 24333969

[10] Peelle JE Troiani V Grossman M Wingfield A. Hearing loss in older adults affects neural systems supporting speech comprehension. J Neurosci. (2011) 31:12638–43. 10.1523/JNEUROSCI.2559-11.2011 21880924 PMC3175595

[11] Fischl B Sereno MI Dale AM. Cortical surface-based analysis. II: inflation, flattening, and a surface-based coordinate system. Neuroimage. (1999) 9:195–207. 10.1006/nimg.1998.0396 9931269

[12] Fischl B Sereno MI Tootell RB Dale AM. High-resolution intersubject averaging and a coordinate system for the cortical surface. Hum Brain Mapp. (1999) 8:272–84. 10.1002/(sici)1097-0193(1999)8:4<272::aid-hbm10>3.0.co;2-4 10619420 PMC6873338

[13] Fischl B Dale AM. Measuring the thickness of the human cerebral cortex from magnetic resonance images. Proc Natl Acad Sci USA. (2000) 97:11050–5. 10.1073/pnas.200033797 10984517 PMC27146

[14] Fischl B van der Kouwe A Destrieux C Halgren E Ségonne F Salat DH. Automatically parcellating the human cerebral cortex. Cereb Cortex. (2004) 14:11–22. 10.1093/cercor/bhg087 14654453

[15] Desikan RS Ségonne F Fischl B Quinn BT Dickerson BC Blacker D. An automated labeling system for subdividing the human cerebral cortex on mri scans into gyral based regions of interest. Neuroimage. (2006) 31:968–80. 10.1016/j.neuroimage.2006.01.021 16530430

[16] Destrieux C Fischl B Dale A Halgren E. Automatic parcellation of human cortical gyri and sulci using standard anatomical nomenclature. Neuroimage. (2010) 53:1–15. 10.1016/j.neuroimage.2010.06.010 20547229 PMC2937159

[17] Uchida Y Nishita Y Kato T Iwata K Sugiura S Suzuki H. Smaller hippocampal volume and degraded peripheral hearing among japanese community dwellers. Front Aging Neurosci. (2018) 10:319. 10.3389/fnagi.2018.00319 30386230 PMC6198789

[18] Profant O Škoch A Tintěra J Svobodová V Kuchárová D Svobodová Burianová J. The influence of aging, hearing, and tinnitus on the morphology of cortical gray matter, amygdala, and hippocampus. Front Aging Neurosci. (2020) 12:553461. 10.3389/fnagi.2020.553461 33343328 PMC7746808

[19] Kaye JA Swihart T Howieson D Dame A Moore MM Karnos T. Volume loss of the hippocampus and temporal lobe in healthy elderly persons destined to develop dementia. Neurology. (1997) 48:1297–304. 10.1212/WNL.48.5.1297 9153461

[20] Churchyard A Lees AJ. The relationship between dementia and direct involvement of the hippocampus and amygdala in Parkinson's disease. Neurology. (1997) 49:1570–6. 10.1212/WNL.49.6.1570 9409348

[21] Jacobs HIL Hopkins DA Mayrhofer HC Bruner E van Leeuwen FW Raaijmakers W. The cerebellum in Alzheimer's disease: evaluating its role in cognitive decline. Brain. (2018) 141:37–47. 10.1093/brain/awx194 29053771

[22] D'Angiolini S Basile MS Mazzon E Gugliandolo A. *In silico* analysis reveals the modulation of ion transmembrane transporters in the cerebellum of Alzheimer's disease patients. Int J Mol Sci. (2023) 24:13924. 10.3390/ijms241813924 37762226 PMC10530854

[23] Allen JS Bruss J Brown CK Damasio H. Normal neuroanatomical variation due to age: the major lobes and a parcellation of the temporal region. Neurobiol Aging. (2005) 26:1245-60. 10.1016/j.neurobiolaging.2005.05.023 16046030

[24] Thompson PM Hayashi KM de Zubicaray G Janke AL Rose SE Semple J. Dynamics of gray matter loss in Alzheimer's disease. J Neurosci. (2003) 23:994–1005. 10.1523/JNEUROSCI.23-03-00994.2003 12574429 PMC6741905

[25] Griffiths TD Lad M Kumar S Holmes E McMurray B Maguire EA. How can hearing loss cause dementia? Neuron. (2020) 108:401–12. 10.1016/j.neuron.2020.08.003 32871106 PMC7664986

