# Supplementary material for: Brain volumetric analysis of elderly patients based on hearing levels quantified using FreeSurfer
Source: Front Neurol. 2025 Jul 23;16:1602383. doi: 10.3389/fneur.2025.1602383 (PMC12326090; doi:10.3389/fneur.2025.1602383)
Supplement: Supplementary file 1 [file Table_1.docx]

**Supplementary Table 1.** Baseline demographic and health characteristics of the study participants categorized into three groups based on their hearing level: normal hearing, mild hearing loss, and moderate-to-severe hearing loss.

| Variable | Normal Hearing  (n = 328) | Mild Hearing Loss  (n = 336) | Moderate-to-severe Hearing Loss  (n = 81) | *p*-value |
| --- | --- | --- | --- | --- |
| **Age (Year)** | 64.63 ± 4.73 | 67.43 ± 5.74 | 73.75 ± 7.22 | **<0.001** |
| **Sex (Male, %)** | 164 (50%) | 188 (55.95%) | 53 (65.43%) | **0.032** |
| BMI | 24.11 ± 2.78 | 24.17 ± 2.71 | 24.28 ± 3.04 | 0.656 |
| HDL | 57.40 ± 14.84 | 55.12 ± 13.89 | 55.95 ± 13.74 | 0.248 |
| **LDL** | 124.61 ± 39.31 | 118.48 ± 39.41 | 112.73 ± 36.09 | **0.021** |
| Triglyceride | 107.77 ± 59.83 | 114.35 ± 66.13 | 97.63 ± 48.01 | 0.157 |
| **Total cholesterol** | 180.05 ± 39.14 | 172.87 ± 39.19 | 165.33 ± 33.78 | **0.004** |
| **Hypertension** | 203 (61.89%) | 221 (65.77%) | 62 (76.54%) | **0.044** |
| **Diabetes** | **46 (14.02%)** | **73 (21.73%)** | **20 (24.69%)** | **0.013** |
| **HbA1c** | **5.77 ± 0.73** | **5.98 ± 0.91** | **6.13 ± 1.10** | **<0.001** |
| **Glucose** | **106.59 ± 22.94** | **111.49 ± 28.03** | **115.26 ± 32.72** | **<0.001** |
| Alcohol consumption dose | 35.03 ± 44.52 | 41.02 ± 53.49 | 28.24 ± 41.81 | 0.189 |
| Alcohol consumption frequency |  |  |  | 0.509 |
| None | 139 (42.38%) | 146 (43.45%) | 44 (54.32%) |  |
| In a month ≤1 | 61 (18.6%) | 61 (18.15%) | 15 (18.52%) |  |
| 2≤ in a month ≤4 | 62 (18.9%) | 54 (16.07%) | 7 (8.64%) |  |
| 2–3 times a week | 44 (13.41%) | 51 (15.18%) | 11 (13.58%) |  |
| 4≤ in a week | 22 (6.71%) | 24 (7.14%) | 4 (4.94%) |  |
| Pack-years | 30.37 ± 18.56 | 32.55 ± 21.12 | 34.04 ± 21.46 | 0.627 |
| Smoking history |  |  |  | 0.107 |
| Never | 193 (58.84%) | 190 (56.55%) | 39 (48.15%) |  |
| Former | 111 (33.84%) | 105 (31.25%) | 34 (41.98%) |  |
| Current | 24 (7.32%) | 41 (12.2%) | 8 (9.88%) |  |

**Supplementary Table 2.** Brain morphometric analysis of auditory groups, which reports brain volume differences in various brain regions between groups with different levels of hearing loss using FreeSurfer analysis.

| Hemisphere | Segmentation area  (mm^3^) | Normal Hearing  (n = 328) | Mild Hearing Loss  (n = 336) | Moderate-to-severe Hearing Loss  (n = 81) | *p*-value |
| --- | --- | --- | --- | --- | --- |
| **Total Gray matter** | | **604532.85 ± 30404.46** | **592999.57 ± 35990.44** | **574038.19 ± 31400.18** | **<0.000** |
| Left | **Cerebellar white matter** | **14231.54 ± 2072.88** | **13977.10 ± 1964.33** | **13356.72 ± 2519.41** | **0.003** |
|  | **Cerebellum Cortex** | **51374.41 ± 4537.37** | **50229.56 ± 4586.75** | **48679.98 ± 4739.08** | **<0.000** |
|  | **Thalamus** | **7366.21 ± 765.51** | **7218.27 ± 725.19** | **7032.60 ± 743.39** | **0.001** |
|  | Caudate | 3316.60 ± 390.17 | 3287.23 ± 472.27 | 3240.07 ± 405.87 | 0.325 |
|  | Putamen | 4543.86 ± 491.93 | 4483.18 ± 578.89 | 4342.69 ± 636.49 | 0.012 |
|  | Pallidum | 1900.63 ± 193.72 | 1878.29 ± 215.78 | 1874.84 ± 239.59 | 0.327 |
|  | **Hippocampus** | **4023.86 ± 434.44** | **3886.94 ± 393.79** | **3675.06 ± 440.67** | **<0.000** |
|  | **Amygdala** | **1558.20 ± 236.29** | **1506.59 ± 214.49** | **1432.58 ± 241.88** | **<0.000** |
|  | **Accumbens** | **412.35 ± 105.35** | **380.12 ± 99.93** | **334.69 ± 94.39** | **<0.000** |
| Right | **Cerebellar white matter** | **13636.49 ± 2216.89** | **13423.76 ± 2064.38** | **12661.41 ± 2398.90** | **0.002** |
|  | **Cerebellum Cortex** | **51958.32 ± 4575.01** | **50711.04 ± 4703.82** | **49471.82 ± 4359.63** | **<0.000** |
|  | **Thalamus** | **6984.98 ± 633.64** | **6856.11 ± 655.02** | **6675.81 ± 612.04** | **0.000** |
|  | Caudate | 3397.56 ± 375.55 | 3411.39 ± 449.72 | 3334.47 ± 515.39 | 0.347 |
|  | Putamen | 4633.78 ± 514.76 | 4579.13 ± 616.63 | 4482.89 ± 649.27 | 0.093 |
|  | Pallidum | 1918.10 ± 207.25 | 1896.65 ± 209.10 | 1891.69 ± 237.79 | 0.351 |
|  | **Hippocampus** | **4242.58 ± 499.99** | **4086.91 ± 444.19** | **3839.33 ± 475.34** | **<0.000** |
|  | **Amygdala** | **1760.00 ± 238.37** | **1724.18 ± 218.94** | **1641.07 ± 248.40** | **0.000** |
|  | **Accumbens** | **469.12 ± 89.19** | **448.86 ± 86.36** | **405.66 ± 101.03** | **<0.000** |

**Supplementary Table 3.** Results of multiple linear regression analyses examining the effect of hearing group on cortical volume across 13 brain regions, including uncorrected and false discovery rate (FDR)-corrected p-values for multiple comparisons.

|  | Segmentation area (mm^3^) | Uncorrected  *p*-value | FDR-corrected  *p*-value |
| --- | --- | --- | --- |
| **Total Gray matter** | | **0.011** | 0.143 |
| Left | Cerebellar white matter | 0.902 | 0.902 |
|  | Cerebellum Cortex | 0.203 | 0.377 |
|  | Thalamus | 0.127 | 0.330 |
|  | Hippocampus | 0.058 | 0.251 |
|  | Amygdala | 0.482 | 0.671 |
|  | Accumbens | 0.106 | 0.330 |
| Right | Cerebellar white matter | 0.599 | 0.671 |
|  | Cerebellum Cortex | 0.163 | 0.353 |
|  | Thalamus | 0.565 | 0.671 |
|  | **Hippocampus** | **0.039** | 0.251 |
|  | Amygdala | 0.619 | 0.671 |
|  | Accumbens | 0.232 | 0.377 |
